# Auditing and instructing text-to-image generation models on fairness

**DOI:** 10.1007/s43681-024-00531-5

**Published:** 2024-08-01

**Authors:** Felix Friedrich, Manuel Brack, Lukas Struppek, Dominik Hintersdorf, Patrick Schramowski, Sasha Luccioni, Kristian Kersting

**Affiliations:** 1 Artificial Intelligence and Machine Learning Lab, Technical University of Darmstadt, Darmstadt, Germany; 2 Hessian Center for Artificial Intelligence (hessian.AI), Darmstadt, Germany; 3 German Research Center for Artificial Intelligence (DFKI), Darmstadt, Germany; 4 LAION, Online, Hamburg, Germany; 5 Huggingface, Montreal, Canada; 6 Centre for Cognitive Science, Technical University of Darmstadt, Darmstadt, Germany

**Keywords:** Fairness, Stable diffusion, Text-guided image generation, Text-to-image synthesis, Model audit, Model debiasing

## Abstract

Generative AI models have recently achieved astonishing results in quality and are consequently employed in a fast-growing number of applications. However, since they are highly data-driven, relying on billion-sized datasets randomly scraped from the internet, they also suffer from degenerated and biased human behavior, as we demonstrate. In fact, they may even reinforce such biases. To not only uncover but also combat these undesired effects, we present a novel strategy, called Fair Diffusion, to attenuate biases during the deployment of generative text-to-image models. Specifically, we demonstrate shifting a bias in any direction based on human instructions yielding arbitrary proportions for, e.g., identity groups. As our empirical evaluation demonstrates, this introduced control enables instructing generative image models on fairness, requiring no data filtering nor additional training.

## Introduction

Artificial intelligence (AI) has become an integral part of our lives. However, the deployment of AI systems has sparked a debate on important ethical concerns, especially around fairness. There is a growing concern that AI systems perpetuate and even amplify existing biases, leading to unfair outcomes. One key area where fairness is critical is text-to-image synthesis [1–5], which has revolutionized a range of applications, including marketing and social media. Diffusion Models (DM), like Stable Diffusion (SD) [1], have recently become a widely used variant of image synthesis models, which generate realistic and high-quality images based on text input.

**Fig. 1:**
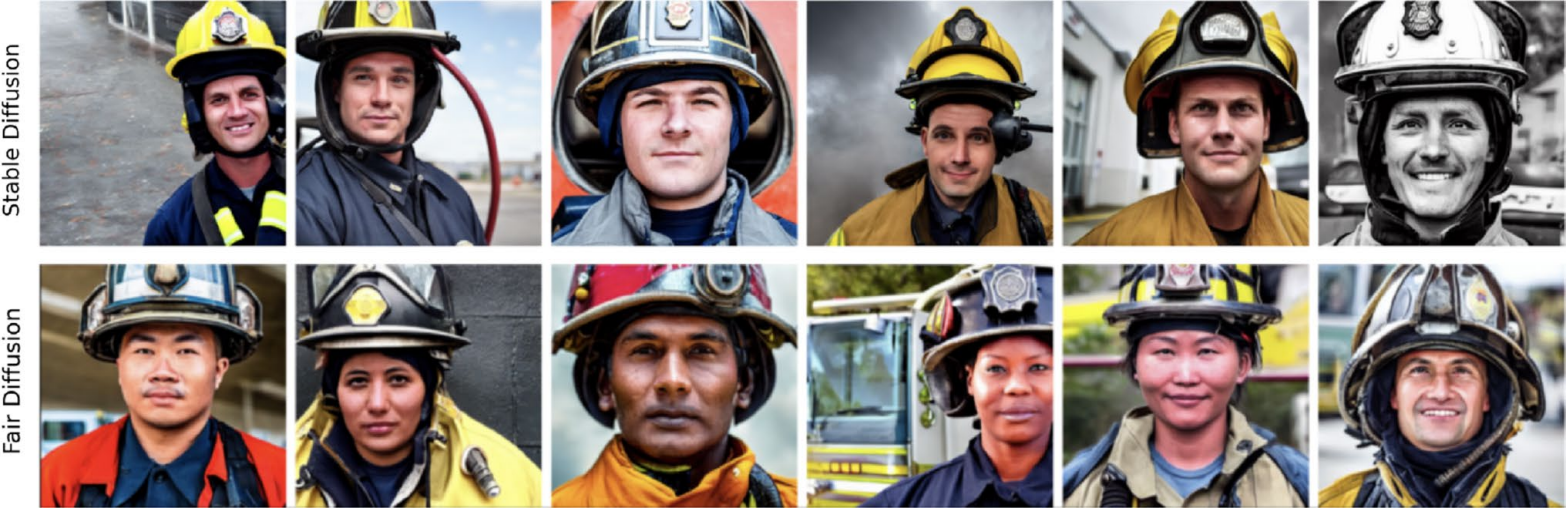
Stable diffusion (top row) runs the risk of lacking diversity in its output (here, e.g., only White male-appearing persons as “firefighters”). In contrast, Fair Diffusion (bottom row) allows one to introduce fairness—increasing outcome impartiality—according to a user’s preferences (e.g., group identities of “firefighters”)

However, despite these successes, they inherently suffer from biased [6–8] and unfair behavior (cf. Fig. 1) similar to generative language models [9]. One particular assertion being made concerns *bias amplification* [8]. In order to understand bias flow within a diffusion model (e.g. bias amplification) it is necessary to audit each model component for bias and compare them. In this regard, our approach is twofold. (i) Therefore, as a means to understand bias flow within a diffusion model, we audit each of their components for biases. (ii) After the audit, we eventually tackle mitigating the found biased behavior. Inspired by advances in instructing AI systems based on human feedback [10, 11], we here explore bias mitigation via the instruction of text-to-image models on fairness. As part of our audit, we evaluate biases in the publicly available text-to-image model SD, its large-scale training dataset LAION (Large-scale Artificial Intelligence Open Network) [12] as well as its pre-trained CLIP (Contrastive Language-Image Pre-Training) text encoder [13]. Therefore, we created a subset of LAION-5B [12] containing over 1.8M images depicting over 150 occupations to approximate the data’s gender occupation bias. On the other hand, we identify potential strategies for addressing these gender biases. To this end, we propose a novel and advanced strategy, Fair Diffusion, to promote fairness (cf. Fig. 1). It utilizes a (textual) interface to instruct the model on fairness during deployment, which we envision as essential for designing and implementing fair DMs.

Fair Diffusion builds on critical concepts captured in a model from its training and steers them in a given direction to increase fairness at inference. The user is put in control and guides the model by instructing it on fairness. For the first time, Fair Diffusion offers a practical approach to fairness in DMs. This way, it is possible to realize different notions of fairness, e.g., outcome impartiality, in a single framework easily accessible to individuals. By addressing these fairness issues, we pave the way for DMs to be used in a way that is fairer and more beneficial to society. More importantly, with our strategy, users regain some control over the model’s output, which has previously been ceded to a small number of entities with large computational resources. To summarize, we contribute by

(i) auditing the components of Stable Diffusion for (gender-occupation) biases to identify potential bias amplification,
(ii) proposing and evaluating a novel strategy, Fair Diffusion, to overcome and mitigate unfair model outcomes,
(iii) discussing future pathways for fair generative image models, specifically how they can be integrated into societies to directly promote fairness with a user in control.

We provide the data and code to reproduce our experiments, enabling model providers to build upon our approach.^1^

The paper is organized as follows. We start off with related work on bias mitigation in large-scale models in Sect. 2. Next, in Sect. 3, we introduce the background and underlying methodology of Fair Diffusion, which enables us to mitigate biases in diffusion models, including the applied definition of fairness. In Sect. 4, we examine the components of Stable Diffusion for biases and demonstrate their mitigation using Fair Diffusion on the example of gender occupation bias. Before concluding, in Sect. 5, we extensively discuss our results and highlight a focal shift in achieving fairness through interaction with biased models during deployment.

**Disclaimer** This paper depicts images of different kinds of biases and stereotypes that some readers may find offensive. We emphasize that the goal of this work is to investigate and eventually mitigate these biases, which are already present in generative models. We do not intend to discriminate against identity groups or cultures in any way.

## Related work on bias mitigation

Recently, many approaches have been proposed to create models with fairness in mind. For large-scale models, these methods can be categorized with respect to three paradigms: (1) pre-processing the training data to remove bias before learning, (2) enforcing fairness during training by introducing constraints on the learning objective, and (3) post-processing approaches to modify the model outcome during deployment.

For the first paradigm, several works [14–16] have focussed on documenting datasets as a predecessor for preprocessing. For example, Yang et al. [14] annotated the ImageNet dataset for protected attributes, whereas Prabhu et al. [15] and Schramowski et al. [16] focus on safety concepts. As a next step, for DMs specifically, Nichol et al.  [4] filtered the data prior to training in order to mitigate bias through the removal of biased data. However, they observed the filtered model continuing to exhibit bias while encountering adverse effects such as a loss in generalization ability. These results highlight that creating a completely bias-free dataset is not feasible. Additionally, different definitions of fairness would each require a dedicated dataset and thus model tailored to the targeted fairness characteristics. This contradicts a major principle of large-scale pre-training, i.e., training one model on only one (large) dataset and subsequently using it for various downstream tasks. Hence, data pre-processing alone does not provide an apt solution for mitigating biases.

Other works follow the second paradigm by optimizing the model’s parameters. Common approaches to debias concepts in a DM are jointly training or finetuning a model with adversarial training [17–19], a distributional alignment loss [20], or time-dependent importance reweighting [21]. Similarly, Zhang et al. [22] employ reinforcement learning approaches to optimize a model for fairness. There are also multiple approaches that learn special *fair* tokens that are appended or inserted to each input prompt in order to debias image generation [23–25]. Lastly, Li et al. [26] optimize the embedding space and Shrestha et al. [27] integrate and train a retrieval mechanism for fair image generation. Yet, all these approaches require many resources (specifically computation, memory, and time) and cannot be applied ad hoc.

In contrast, our work targets the (post-process) deployment stage of DMs, i.e. the third paradigm. Fortunately, large-scale models are not at the mercy of under-curated data. Schramowski et al.  [6] demonstrated that biased representations learned during pre-training can be exploited to suppress unwanted and inappropriate behavior in the downstream task. While their work focused on suppressing inappropriate content like pornography, we here focus on *fair* outcomes. In general, several image guidance and editing techniques during deployment [28–31] have been proposed.^2^ In this work, we employ a guidance technique similar to Sega [28]. With this tool at hand, users can instruct a model on their individual definition of fairness. Previous studies have already shown that such user instructions are an essential component for machine learning models to enable user alignment [10, 33, 34], trust [35], and overall model performance [11].

## Fair diffusion

Before we examine biases in text-to-image models and corresponding mitigation strategies, let us present a novel strategy, Fair Diffusion. To this end, we propose to instruct text-to-image DMs on fairness with textual guidance. First, we generally explain image generation with textual guidance. Next, we elaborate on fairness definitions in the scope of investigating DMs. Finally, we devise our new fairness strategy as well as means for audit and evaluation.

### Text-guided image generation

**Fig. 2:**
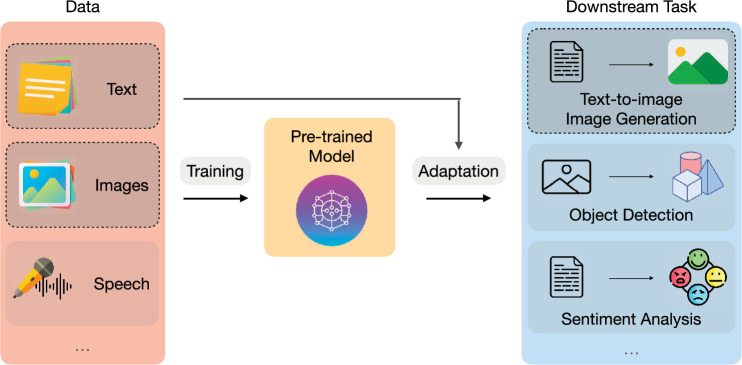
Setup of large-scale AI models. Many recent AI systems are centered around a pre-trained model [36]. For one, this model is pre-trained on large-scale data, often multimodal. On the other hand, it is adapted to a downstream task, e.g., by fine-tuning. This work focuses on DMs, and for many of them, CLIP serves as the pre-trained model, which is trained on text and image data (dashed boxes, left). It is adapted for the downstream task such that only its text encoder is integrated into the DM to generate images from this text (dashed box, right). In turn, the DM is adapted to the downstream task by fine-tuning on task-specific data. (Best viewed in color) (color figure online)

As visualized in Fig. 2, many recent models, like DMs, train on large-scale datasets and additionally incorporate other large-scale pre-trained models. These are important aspects to perform well on text-to-image generation tasks and generalize over multiple domains. The information transfer from the pre-trained model and the downstream adaptation in DMs helps these models achieve remarkable performance. However, both components will introduce biases into the models, as we demonstrate in our experiments.

The underlying intuition of DMs for image generation is as follows: the generation starts from random noise *z*, and the goal is to remove this noise in order to obtain a high-quality output. The DM calculates an estimate of the current noise $\tilde{\epsilon }$ in an image. Subtracting this noise estimate from the initial noisy image results in a high-fidelity noise-free image $x = z - \tilde{\epsilon }$. Since predicting this noise is a hard problem, multiple denoising steps *T* are applied iteratively. In each step, the current noise is estimated, of which a small amount $\tilde{\epsilon _t}$ is subtracted, approximating the overall $\tilde{\epsilon }$.

1 $$\begin{aligned} z_{t+1}&= z_t - \tilde{\epsilon _t} \,. \end{aligned}$$

The final image *x* is equivalent to the last iteration of denoising steps, $x = z_T$. For text-to-image generation, the model’s $\tilde{\epsilon }$-prediction starts from random noise $z_t$ and is conditioned on text-prompt *p*, which is encoded to $c_p$, overall resulting in a generated image faithful to that prompt

2 $$\begin{aligned} \tilde{\epsilon _t}&= \text {DM}_{\theta }(z_t, c_p) = \tilde{\epsilon _\theta }(z_t, c_p) \,. \end{aligned}$$

The textual interface, i.e. text conditioning, is realized through classifier-free guidance [37], the standard technique for current diffusion models. In more detail, during image generation, the unconditioned noise prediction $\tilde{\epsilon _\theta }(z_t)$ is pushed in the direction of the text-conditioned $\tilde{\epsilon _\theta }(z_t, c_p)$ to yield an image aligned with prompt *p*. For the interested reader, more details on the general function of diffusion models can be found at [38]. As we will show, we improve this text conditioning to steer the generated image toward fairer outcomes by leveraging multiple text instructions.

### Fairness for diffusion models

Fairness has always been a challenging concept to define [39, 40]. Definitions of fairness and bias, like many ethical concepts, are always controversial, resulting in many valid definitions, as discussed by many [39–42]. Roughly, fairness can be summarized as the absence of any tendency in favor of a person due to some attribute. However, fairness is inherently subjective and overall incomplete. In general, only in very specific and constrained situations, it is possible to satisfy multiple of these fairness notions. In turn, a universal definition is not available as investigated by previous works [40, 43–45]. We define fairness for Fair Diffusion, in line with closely related work on fairness [46], as algorithmic fairness for a dataset and model.

#### Definition 1

Given a (synthetic) dataset $\mathcal {D}$, fairness or statistical parity is defined as

3 $$\begin{aligned} P(x,y = 1|a = 1) = P(x,y = 1|a = 0) \,. \end{aligned}$$

Here, $y\in \mathcal {Y}$ is the label of a respective data point $x\in \mathcal {X}$, *a* is a protected attribute and *P* is a probability. For example, *x* can be an image with the label *y* “firefighter” and *a* the protected attribute “gender”. Definition 1 can be used to evaluate the fairness of a dataset but also a generative model. Typically, datasets consist of real-world data *x* with human labels *y*. For a generative model, a dataset can be synthetically generated to enable an empirical fairness evaluation. In that case, a data point is obtained through $x=\eta (y)$, where the model $\eta$ is prompted by the user with the desired text label *y*. The model $\eta$ can represent any generative downstream task for any input modality (visual, textual, etc.). For instance, we consider $\eta$ as a generative DM mapping from text (also called prompt *p*) to images, $x=\eta (p)$. In other words, we define a dataset to be fair if Definition 1 holds, i.e., there is no disproportionate weight in favor of attribute *a* in the data. Similarly, we define a model to be fair if the same holds for a model’s generated output (e.g., images). The given definition ensures fairness for a binary attribute but can be generalized to multiple non-binary attributes. Yet, they may interfere with each other such that it becomes more challenging to satisfy them at the same time. Furthermore, this fairness definition requires all attributes to be known, definable, measurable, and separable. We discuss the limitations of this definition later (cf. Sect. 5). To ensure statistical parity (Definition 1), both attribute expressions must be represented equally in the model’s outcome. This results in a uniform probability distribution, assigning the same probability to each expression of an attribute, i.e. $P(a) = \frac{1}{|a|}$.

**Fig. 3:**
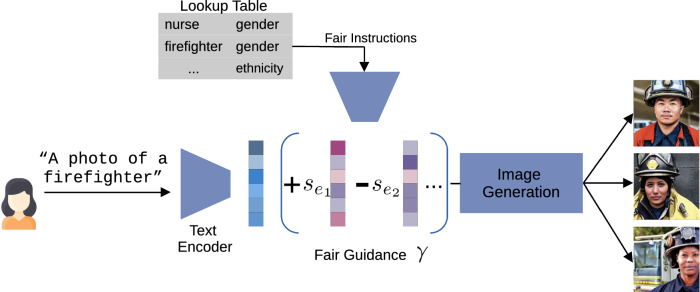
Fair Diffusion deployment. A user inserts a prompt to generate an image. With the help of fair guidance, image generation is steered toward a fairer outcome. Here, the fair instructions are realized with a lookup table: the biased concept is recognized, and thus guidance $\gamma$ is applied. Like in Eq. (5), the fair instructions $e_i$ are transformed into vectors $c_{e_i}$ by the text encoder and can be scaled by $s_{e_i}$. Here two editing prompts (purple-colored vectors) are illustrated. The lookup table can be set up by any user. (Best viewed in color) (color figure online)

### Instructing text-to-image models on fairness

With a definition of fairness established, the next step is to actually enforce and maximize it during image generation. Let us now understand the general setup of Fair Diffusion (cf. Fig. 3) before delving into the technical details.

In line with the goal of instructing DMs on fairness, previous work has proposed approaches to control image generation. While Fair Diffusion can, in principle, utilize any of these techniques, we here evaluate Fair Diffusion with Semantic Guidance (Sega [28]). Sega extends the image generation with additional textual guidance terms which enable flexible image manipulations. This way, certain concepts in an image can be changed with high precision and quality. Building on this tool, we can guide a model to promote fairness and reduce bias in its outcomes through additional text conditioning. Intuitively and on a high level, Fair Diffusion extends the standard image generation with an additional fair guidance term $\gamma$ (cf. Eq. 5). Fair guidance can be seen as an additional control tool, which aligns the model outcome with the users’ fair instructions. We generate an image with

4 $$\begin{aligned} x = \eta (p,\gamma ) \,. \end{aligned}$$

This way, the image generation $\eta$ is a function of text input prompt *p* and fairness conditioning $\gamma$. In turn, $\gamma$ depends on additional textual descriptions of attribute expressions $e_i$, with guidance scale and direction $s_{e_i}$. The direction of the fair guidance is randomly switched based on *P* (Eq. 3), in order to realize the different expressions of one attribute. Consequently, each $e_i$ is either increased or decreased depending on the expression that should be promoted/ suppressed. Fair Diffusion supports arbitrary distributions *P* enabling the implementation of different definitions of fairness. For the evaluation, we verify the resulting attribute distributions with a classifier. Ideally, the user-defined and measured distributions match.

Figure 3 illustrates a user generating images displaying “firefighters” with Fair Diffusion. Fair guidance helps to generate more diverse “firefighters”. In this binary example, concept $e_1$ is promoted ($+$) and $e_2$ suppressed (−) during image generation. For instance, the generation is conditioned on $+$“female” and −“male”, yielding a “female”-appearing “firefighter”. Here, a lookup table serves as an automatic means to identify text prompts requiring fair guidance. It contains fair instructions that realize fair guidance to align the output with users’ fairness notions. This allows Fair Diffusion to be automatized and integrated into deployed models and their APIs.

**Fair guidance** In the following, we explain image generation with fair guidance in more detail. In addition to text prompt *p*, fair guidance is provided via textual attribute descriptions $e_i$ (with their own scale and direction). To this end, we extend standard text-guided image generation from Eq. (2) with fair guidance, resulting in Eq. (5). This extends the previous noise estimation for image generation from $\bar{\epsilon }_\theta (z_t, c_p)$ to $\bar{\epsilon }_\theta (z_t, c_p, c_e)$. This way, the image generation is conditioned on the normal input prompt (classifier-free guidance) and additionally on the fairness instructions (fair guidance). The resulting $\epsilon$-estimate can be written as

5 $$\begin{aligned} \bar{\epsilon }_\theta (z_t, c_p, c_e)= \underbrace{ \epsilon _\theta (z_t) + s_g \big (\epsilon _\theta (z_t, c_p) - \epsilon _\theta (z_t)\big )}_{\text {classifier-free guidance}} + \underbrace{\gamma (z_t, c_e, s_e)}_{\text {fair guidance}} \end{aligned}$$

where *c* represents the encoding of each textual input. This means in the context of DMs, as detailed in Sect. 3.1, during each iteration of the diffusion process, we calculate multiple noise estimations (unconditioned, input prompt and fair guidance prompts). These estimations are merged using percentile thresholding to produce a new, *fairer* noise estimation. Moreover, fair guidance can incorporate multiple arbitrary attributes that can be either increased or decreased during image generation. Consequently, more complex changes can be realized by, e.g., guiding toward the encoding $c_{e_1}$ of one concept $e_1$ and simultaneously away from $c_{e_2}$ for concept $e_2$, e.g., its alleged opposite. We describe further details on this design choice in Appendix Fig. 13.

### Auditing the components of diffusion models for fairness

Previously, we showed that DMs are built around various components (cf. Fig. 2) and each can be affected by bias. Namely, bias in the data, the pre-trained model, and its reflection in the downstream task. Next, we describe the measures we employed to quantify and track biases across all three components. This is relevant to understanding bias amplification, e.g. biases in the data get amplified in the downstream task (generated images).

**Data** The first potential source of bias, according to the general model setup (cf. Fig. 2), is the dataset. Given a potentially biased attribute, e.g., gender, we investigate its co-occurrence with a target attribute such as occupation. If, for example, the proportion of genders within all samples of an occupation is not in line with the considered fairness definition (e.g., Definition 1), we have identified a source of bias already emanating from the dataset. This proportion also serves as a reference to investigate whether the model outcome subsequently reflects, amplifies, or mitigates the observed data bias. If the investigated dataset has no pre-existing labels for the attribute(s) of interest, they have to be derived first. This is generally a non-trivial task and cannot easily be transferred between different domains. For vision-language tasks, a sensible approach is to employ a multimodal model capable of computing text-to-image similarity [13]. We identified relevant images $\mathcal {R}$ in the dataset by computing their similarity to a textual description *p* of the target concept. Consequently, the label *y* corresponds to the textual description *p* (e.g., “firefighter”).

In this work, we identified images aligned with description *p* by filtering the entire dataset with an empirically determined similarity threshold $\delta$:

6 $$\begin{aligned} \mathcal {R} = \{i \,\, | \,\, sim(i, p) > \delta \,\, \text {and} \,\, i \in \mathcal {I} \} \end{aligned}$$

where $\mathcal {I}$ denotes the set of all images from the dataset. Next, we used a pre-trained classifier $\kappa$ to determine the (missing) label for the protected attribute under investigation (e.g., “male”-appearing). Accordingly, we obtained each label $a_r$ for image $r\in \mathcal {R}$ with:

7 $$\begin{aligned} a_r = \kappa (r) \end{aligned}$$

**Pre-trained model** Second, we investigated the bias of learned representations in the pre-trained model using the image Embedding Association Test (iEAT) [47]. Intuitively, iEAT tests for statistically significant associations between sets of representations, e.g., encoded images. These consist of two attribute sets *A* and *B* and two target sets *K* and *L*. A common example is target images of female-appearing people *L* and male-appearing people *K* compared against images related to attribute career *A* and family *B*. This way, a biased model may associate the images of male-appearing people closer to “career” than to “family” and vice versa. Formally, the test statistic can be computed as:

8 $$\begin{aligned} s(K,L,A,B) = \sum _{k\in K}s(k,A,B) - \sum _{l\in L}s(l,A,B) \end{aligned}$$

where

9 $$\begin{aligned} s(w,A,B) = mean _{a\in A}cos(w,a) - mean _{b\in B}cos(w,b) \,. \end{aligned}$$

This way, *s*(*w*, *A*, *B*) computes the association of an encoded image *w* with the attributes (*a* and *b*) and eventually the differential association of the encoded target images with the attributes. We assess the statistical significance by computing the one-sided *p*-value along with the effect size *d* as:

10 $$\begin{aligned} p&=Pr_i[s(K_i,L_i,A,B)>s(K,L,A,B)] \end{aligned}$$

11 $$\begin{aligned} d&=\frac{ mean _{k\in K}s(k,A,B) - mean _{l\in L}s(l,A,B)}{\sigma _{w\in K \cup L}(s(w,A,B))} \end{aligned}$$

where $\sigma$ denotes the standard deviation.

**Downstream task** The third source of bias we inspected is the downstream task approximated by its outcome. The outcome modality generally depends on the type of model and task. Here, we evaluated images generated by a diffusion model. The procedure to inspect these images for bias is similar to the dataset inspection: a synthetic image dataset is created, and attribute correlations in it are calculated which are in turn evaluated for fairness, e.g., according to Definition 1. To investigate potential bias transfer between the training data and outcome (mitigation, reflection or amplification), we generated images using the same text prompt *p*, used to search the dataset. Similarly, we used the same classifier $\kappa$ (Eq. 7) to determine label $a_g$ of the protected attribute in the generated images ($g\in \mathcal {G}$) with $a_g = \kappa (g)$. The outcome of the downstream task is of particular interest as it reveals how it mirrors or amplifies biases inherent in its foundational components.

## Experiments

In this section, we first describe experimental details. Then we investigate the components of Stable Diffusion for bias on the prominent example of gender occupation biases. Subsequently, we demonstrate the mitigation of these biases using Fair Diffusion.

### Experimental protocol

For assessing the bias of text-to-image DMs and its mitigation, we inspect the publicly-available diffusion model Stable Diffusion v1.5 (SD [1]), its underlying large-scale dataset (LAION-5B [12]) and pre-trained model (CLIP [13]). Our instruction tool is built around Sega [28] to edit images and guide the image generation toward fairer outcomes and we employed FairFace [48] as $\kappa$ to derive the protected attribute, i.e. facial (gender) attributes. Yet, Fair Diffusion can in principle facilitate any image editing and classifying tools. We show further experimental details in Appendix D.

**Fig. 4:**
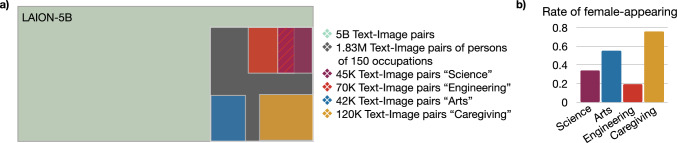
Bias inspection in LAION-5B. **a** Proportion of evaluated images. In total, we identified 1.83M images for gender occupation bias. We built four exemplary subsets (“Science”, “Arts”, “Engineering”, and “Caregiving”) of the occupation set to gain first insights into present biases. Some sets overlap (hatched) as the concepts are not disjunct. Sizes are only illustrative, and actual numbers are given in the legend. For the final evaluation, we use all 1.83M images (gray). **b** Bias evaluation. The “Science” and “Engineering“ subsets have lower rates of female-appearing persons, while “Arts” and “Caregiving” have higher rates of female-appearing persons. Consequently, the inspected LAION-5B images represent stereotypical gender-occupation biases. (Best viewed in color) (color figure online)

**Prompt design** We employed CLIP to identify relevant images in LAION-5B—i.e., depicting people in recognizable occupations—and computed text-image similarities between LAION-5B images and a text prompt representing an occupation. To this end, we used $p=\text {``A photo of the face of a} \{\texttt {occ}\}''$ as a text prompt and empirically determined a similarity threshold $\delta =0.27$. We also used this prompt to generate images with SD, where $\texttt {occ}\in \{\text {``firefighter'', ``teacher'', ``aide'',...}\}$. The whole list consists of over 150 different occupations^3^ and we generated 250 images for each occupation prompt. With this approach, we created a new subset of LAION-5B by identifying over 1.83 million images displaying humans with recognizable faces and in recognizable occupations (cf. Fig. 4a). Furthermore, we generated over 37,500 images (150 prompts $\times$ 250 images per prompt) each with SD and Fair Diffusion, respectively. In total, we evaluated more than two million images^4^ for occupation biases.

**Fairness assumptions** We assumed statistical parity to be fair, i.e. that an equal proportion of female- and male-appearing images is desired as derived from Definition 1. Unfortunately, our evaluation is limited by current datasets and derived classifiers (like FairFace) facilitating only binary-valued gender classification, whereas gender is clearly non-binary [49] (extensively examined in Sect. 5). For this research, only “male” and “female” are considered. Interestingly, Fair Diffusion is independent of this evaluation limitation and can, in principle, be applied to non-binary identities (see results on multi-ary ageism). According to *QueerInAI* [49], the availability of data must be improved to represent the diversity of people and thus Promote fairness for the non-binary gender. Therefore, we urge the community to collect more diverse data beyond binary gender. Lastly, we employed a fair boundary (i.e., allow for a deviation of $\pm 4\%$) to soften the binary theoretical assumption. This way, we try to account for natural non-binarity, e.g., the continuous spectrum of gender with its diversity and non-equal birth rate.

**Statistical measures** We computed a per-group statistic (binary “fe/male”-appearing) to further insight into the overall gender occupation bias in each component. Therefore, we divided the list of occupations into f and m, where the f-group denotes more female-biased occupations and the m-group otherwise. If the rate of female-appearing persons in LAION is $> 0.5$ we use f and otherwise m, respectively. Subsequently, we evaluate these lists for each component and generate respective box plots. Without this group distinction, the average bias lies within the fair boundary (although the box plot shows high variance) as there are strong biases in both directions, which cancel each other out in an overall mean computation.

### Auditing stable diffusion for fairness

We start our empirical study by illustrating the presence of biases in the components of SD. This, in turn, lays the foundation for the subsequent bias mitigation. To this end, we audited SD’s three components for gender occupation biases: (1) its training data LAION-5B, (2) its pre-trained model CLIP, and (3) SD’s outcome, i.e. the generated images.

**Table 1:** Bias inspection for CLIP

Topic	Target concept	Attribute concept	p ($\pmb {\downarrow }$)	d ($\pmb {\uparrow }$)
Gender	Male–Female	Science–Arts	0.003	0.63
Gender	Male–Female	Engineering–Caregiving	0.005	0.57
Gender	Male–Female	Career–Family	0.01	0.58
Ethnicity	White Male–Black Female	Science–Arts	0.05	1.48
Ethnicity	White Male–Black Female	Engineering–Caregiving	0.05	1.57
Ethnicity	White Male–Black Female	Career–Family	0.1	0.99

We examine the iEAT for gender occupation biases. The table shows that such biases are present in CLIP, i.e., male-appearing are considered to be closer to career, science, or engineering, compared to female-appearing who are closer to family, arts, and caregiving. All examples have a high effect size, *d*, and are highly significant, i.e., $p \le 0.05$. Furthermore, we evaluated intersectionality biases and found that skin color attributes amplify gender occupation biases

**Uncovering biases in the data and model foundations of stable diffusion** To begin with, we evaluated LAION-5B on four subsets of our occupation list (cf. Fig. 4a), where each subset contains images of occupations belonging to one field (science, arts, engineering, and caregiving)^5^. Therefore, we classified the images of each subset for gender to obtain insights into the rate of female-appearing persons^6^ as a measure of gender occupation bias. Figure 4a shows that LAION-5B contains several occupation biases. One can observe that the rate of female-appearing persons is higher for occupation fields like arts or caregiving. On the other hand, the rate is lower for science or engineering. Both demonstrate stereotypical proportions in the dataset. For inspecting CLIP, we performed a bias association test, the iEAT. In this experiment, we tested the similarity between encoded images of different concepts. In the spirit of Steed et al.  [47], we applied their setup to the CLIP encoder and uncovered similar gender occupation biases (cf. Table 1). For instance, encoded images of male-appearing persons are closer to engineering-related images than encoded images of female-appearing persons, which are, in turn, closer to caregiving-related images. Even worse, we found a bias amplification when the association test on gender occupation bias is modified by ethnic attributes. In this case, images of male-appearing people are represented by European-American appearance and images of females by African-American appearance. Accordingly, an intersectionality bias [50] is present in CLIP too, amplifying the gender occupation bias. We show more details on this experiment in Appendix Table 2. Overall, we found evidence for gender occupation bias in both model components.

**Fig. 5:**
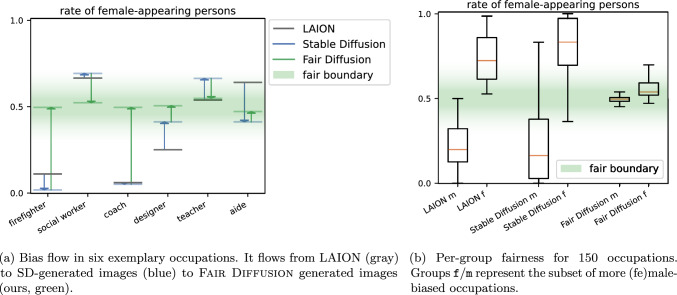
Fairness evaluation for **a** six exemplary and **b** all 150 occupations. **a** For specific occupations, the bias in LAION (gray bar) is sometimes within/outside the fair boundary. The same applies to SD-generated images (blue bar). Blue arrows indicate the bias reflection from LAION to SD images. They do not show a clear tendency for bias amplification. Nonetheless, Fair Diffusion (green) shifts the gender proportion always within the fair boundary. **b** For the full set, LAION and SD-generated images are strongly biased with SD being more biased than LAION. Fair Diffusion mitigates bias toward the fair boundary. Graphs show the rate of female-appearing persons, 1.0 indicates only and 0 no female-appearing persons, whereas 0.5 indicates $50\%$ are female and $50\%$ male appearing. A rate toward the middle is preferred (i.e. fair boundary $50\% \pm 4$). (Best viewed in color) (color figure online)

**Uncovering biases in the outcome of stable diffusion** Next, we examine the third model component, the downstream task, and its outcome, for gender occupation biases. Here, we evaluated the generated images from SD. Since SD builds on LAION-5B, we also compared these respective rates to SD’s outcome for inspecting mirrored unfairness. Figure 5a depicts the rate of female-appearing persons for six exemplary occupations. One can observe that the SD-generated images (blue lines) are clearly gender biased for various occupations. For instance, a firefighter or a social worker are significantly affected. The evaluated LAION-5B images (gray lines) contain similar biases, providing further evidence for our previous findings. However, one can observe a discrepancy in gender occupation biases, e.g., for “firefighter”, between LAION-5B and SD-generated images. The rate of female-appearing persons is higher for the generated images than for its training data, representing a stronger gender bias. On the other hand, if we look at “coach”, the gender bias in the SD outcome is on par with LAION-5B. Furthermore, for a designer, the gender bias in the generated images is smaller than in its training data. At the same time, one can also see that there are occupations like “teacher”, which have nearly no gender bias in LAION-5B (applicable for overall 5% of the evaluated occupations). Lastly, one can also observe that the gender bias in the generated images for “aide” is smaller than in LAION-5B but beyond the fair boundary in the opposite direction. In conclusion, SD’s outcome is clearly biased to a varying extent depending on the occupation and we show evidence for bias amplification, mitigation, and reflection.

**Are biases mirrored between LAION-5B and the outcome of stable diffusion?** Let us now build on the previous anecdotal investigations and examine bias reflection in depth. To this end, we turn from exemplary occupations to the complete occupation subset of LAION-5B (cf. green in Fig. 4a). For the full set, we found that LAION-5B’s gender biases get amplified in the generated images for 56%, reflected for 22%, and mitigated for 22% of the evaluated occupations.^7^ To further insight these different bias reflection behaviors, we computed a per-group statistic (binary fe/male: f/m) to better understand the average gender bias in each component, depicted in Fig. 5b. One can observe that the median (orange line) of SD-generated images and the inspected LAION-5B images are distinctly outside the fair boundary. This means LAION-5B and the SD-generated images are unfair according to Definition 1. More importantly, the median of SD-generated images is farther away from the fair boundary (middle) for both groups (f/m)^8^ compared to LAION-5B. This experiment provides evidence that SD-generated images are on average more unfair than LAION-5B images, further indicating a bias amplification. Particularly, one can observe high variance, urging more research in this direction. Furthermore, attributing the discrepancy in bias to a specific component of the model or aspect of the training procedure is difficult. The shift in bias results from a complex interplay between training data and objective, and CLIP’s inherently biased representations, which are in turn influenced by a different training set.

During our inspection, we found biases and unfairness in each component of the SD pipeline: in the training data (LAION-5B dataset), the foundation model (CLIP encoder), and the outcome (SD-generated images). At the same time, the biases are not simply mirrored between LAION-5B and SD-generated images and do not show a clear tendency.

### Instructing on fairness with Fair Diffusion

After discovering several biases in SD’s components, we turn to mitigate them. Since the interplay between SD’s components is complex, debiasing them is a challenging task. In the following, we evaluate the guidance toward fairness of text-to-image generations.

**Setting up** Fair Diffusion In contrast to the image generation with default SD, we further conditioned the image generation with Fair Diffusion. To this end, we steered the image generation toward “female person” ($e_1$) and away from “male person” ($e_2$) and randomly switched the direction toward male-appearing and away from female-appearing with a 50% chance. This way, we utilize the concepts encoded in a DM to simultaneously suppress one and reinforce the other, with alternating directions. Due to this approach, 50% of the images should contain “fe/male”-appearing generated persons.^9^ For evaluating the impact of fair instructions, we applied Fair Diffusion to SD-generated images, i.e., we re-generated an image with the same seed and parameters and included the additional conditioning (fair guidance $\gamma$) for gender.

**Mitigating gender bias in the outcome of stable diffusion** Figure 5 demonstrates Fair Diffusion’s performance (green line) in mitigating gender occupation biases detected in Stable Diffusion (blue bar). Looking at the six exemplary cases (Fig. 5a), one can observe a shift of the gender proportion to the inside of the fair boundary, regardless of the direction in which the bias was previously present. Furthermore, Fair Diffusion addresses the biases, regardless of whether they were present in LAION-5B or the SD outcome. For example, though the proportion in SD-generated images for “designer” is less biased than in LAION-5B, it is still not within the fair boundary. In turn, Fair Diffusion mitigates the bias further and shifts the gender proportion within the fair boundary. Moreover, the per-group (f/m) median for all occupations is within the fair boundary, so Fair Diffusion substantially reduced unfair gender-occupation proportions (Fig. 5b). Hence, on average, Fair Diffusion achieves a fair model outcome according to Definition 1. Yet, one can see that there remains variance in the generated images for some occupations. This can generally be due to the non-binary nature of gender, and gender is also not to be determined simply based on outward appearance. Moreover, we could identify some outliers, e.g., images of “dishwasher” were generally difficult to generate but also difficult to edit for gender, as it does not only describe an occupation but also a cleaning device. When searching LAION-5B^10^ for “a photo of the face of a dishwasher”, we also mainly found images of the cleaning device and no humans. So, we assume this to be an artifact due to the ambiguity of “face of a dishwasher”. For a broader evaluation, in particular, on the design of the editing prompts, we refer to Appendix C.

**Fig. 6:**
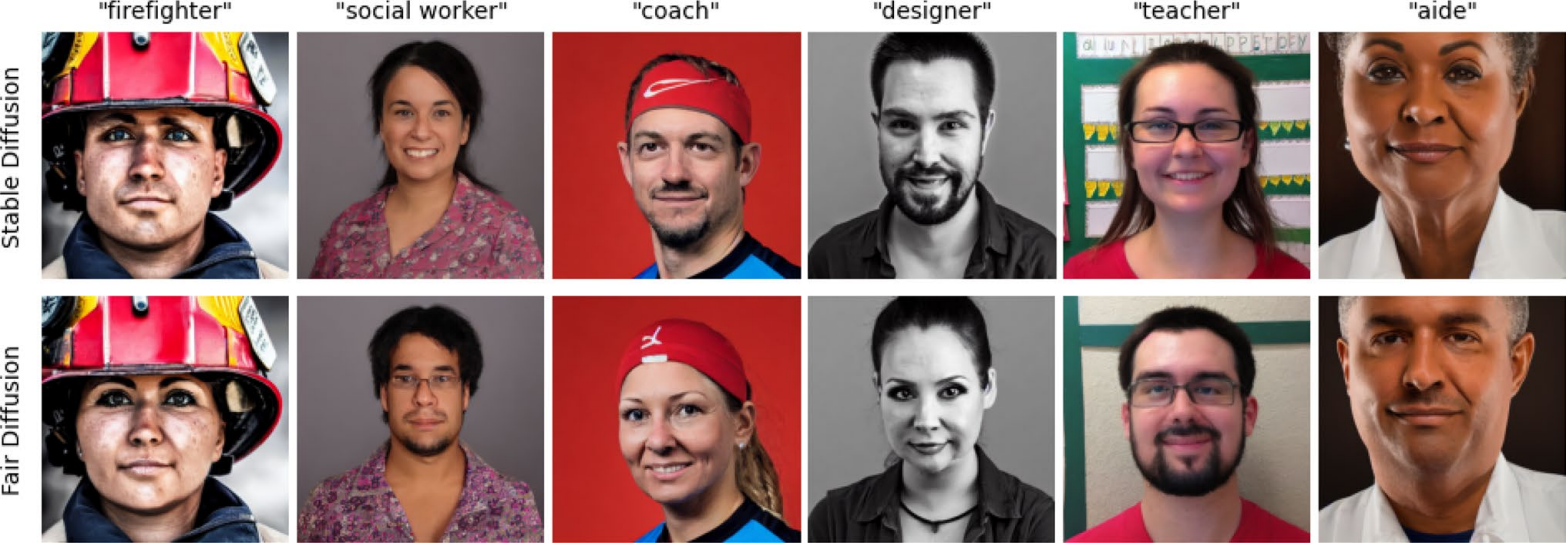
Generated images with SD (top row) and Fair Diffusion (bottom row) for different occupations. The images are generated with the prompt “A photo of the face of a {occ}”, in which each column represents the used occupation (occ). For generated images of female-appearing persons, we applied fair guidance with − “female person” + “male person” and vice versa for male-appearing persons. One can observe that Fair Diffusion changes the typical gender appearance for each occupation image while keeping the residual (occupation-related) features present

Apart from our quantitative analysis, we also show qualitative results on our bias mitigation approach in the following. In Fig. 6, one can observe a shift in outward appearance. The top row shows images generated with SD and prompts *p* for each of the six exemplary occupations. As our inspection experiments already demonstrated, there is mostly a strong bias toward one gender for certain occupations. In contrast, the images generated with Fair Diffusion (bottom row) shift the gender appearance toward the other gender appearance and by that ultimately toward a more diverse output. Notably, the overall image composition remains the same, with only minor changes to the rest of the image, which avoids unnecessary confounding.

**Approaching bias beyond gender** Fair model outcomes are not restricted to gender appearance. In Fig. 1, we used Fair Diffusion to edit multiple features of firefighters’ outward appearance. The result is a more diverse output of facial features regarding gender, skin tone, and ethnicity. These examples demonstrate that Fair Diffusion can independently address multiple biases and increase outcome impartiality beyond one attribute. In Appendix A, we show further qualitative results on biases beyond gender, e.g., heteronormativity and ageism.

**Fig. 7:**
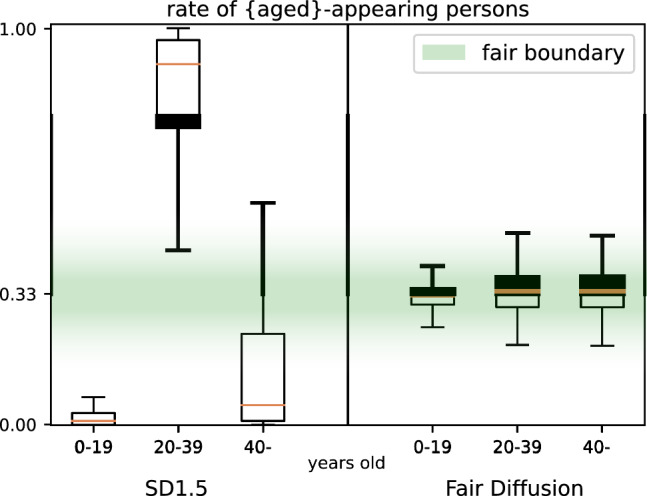
Fair Diffusion vs. Stable Diffusion 1.5 on multi-ary attribute (age-occupation) fairness. Rate of aged-appearing persons for all 150 occupations is given, where $\texttt {aged}\in \{\text {0--19, 20--39, 40--}\} \text {years-old}$. 1 indicates only and 0 no aged-appearing persons. Here, a rate within the fairness boundary ($33\% \pm 4$) is preferred, such that each of the three attributes is equally present. SD-generated images are strongly age-biased for all three age groups, whereas Fair Diffusion shifts this age bias within the fair boundary. (Best viewed in color) (color figure online)

Furthermore, we illustrate Fair Diffusion beyond one binary attribute fairness in Fig. 7. For this setting, we again generate images for our occupation list, but instead of approaching gender occupation bias, we now target ageism. We discretize age into three categories {0–19, 20–39, 40–}. To this end, we employed FairFace again to evaluate the generated images for age appearance. Figure 7 shows that Stable Diffusion 1.5 has a strong bias of generating images of middle-aged persons (20–39 years old) while other age groups are strongly underrepresented. This emphasizes once more the bias of current generative models to generate people of a certain outward appearance (e.g., persons between 20 and 39; young adults) and that these models are very limited in producing diverse outputs. With Fair Diffusion, in contrast, we apply fair guidance to mitigate age bias within the fair boundary. The images generated with Fair Diffusion represent each of the three age groups equally (according to Definition 1). Consequently, we can approach fairness beyond gender and binary attributes. Further experimental details can be found in Appendix D.

In summary, it remains difficult to create a model satisfying fairness in all aspects. Here, we first investigated the components of Stable Diffusion for gender-occupation biases and subsequently approached their mitigation. In this regard, we evaluated instructing text-to-image models with Fair Diffusion to approach outcome impartiality. Our empirical results demonstrated its potential as a reliable approach for gender- and age-occupation bias. Yet, we emphasize the interplay between different debiasing techniques. Ultimately, we envision a future with models that can generate a more diverse outcome in the first place—hand in hand with a user in control.

## Discussion

We have found several severe biases in all components of the SD pipeline and introduced Fair Diffusion to mitigate these biases. Still, we need to delve deeper into some of the insights gained and use this section to discuss them in greater detail. In particular, we touch upon risks and opportunities for society.

**Shifting the debiasing paradigm** This work provides food for thought on current debiasing techniques, mostly focused on dataset curation and in-process bias prevention, now shifting the focus to the deployment stage. As Nichol et al.  [4] showed, curating datasets by filtering has drawbacks, such as persisting biases and worse generalization capabilities. In turn, Fair Diffusion operates at the deployment stage enabling fair outcomes according to Definition 1. On the other hand, our inspection also showed that the evaluated images from LAION-5B are, on average, still remarkably affected by bias. Consequently, debiasing it might remain important. Ultimately, we believe debiasing all components might be necessary to increase fairness in generative models further. Nevertheless, as long as this is not available, especially for end-users, we demonstrated that the text interface of DMs enables instructions as an easy-to-use technique that can be immediately deployed to mitigate biases in the outcome of current image generation models. This does not introduce an entirely *fair* model but instead a way to control unfair models to increase fairness. In the presented version, Fair Diffusion is applied regardless of, e.g., existing biases in LAION-5B. While Fair Diffusion enables one to take control of fairness, a future vision for even fairer models is automated detection of unfairness. If biased concepts are known beforehand, one could supply Fair Diffusion with these in order to take action without one actively intervening in the moment of generation. For example, the lookup table and instructions in Fig. 3 could be filled out beforehand.

**Is the model finally fair?** As shown, Fair Diffusion promotes fairness according to Definition 1. However, as discussed before, fairness is inherently incomplete, such that the setup used in this work does not account for other fairness definitions. For example, it approaches fairness in the model outcome but not in the dataset. To achieve such fairness other techniques must be applied, e.g., modifying the dataset. Still, Fair Diffusion can be used to realize different notions of fairness. If the desired output proportion differs from an equal proportion (50/50 for binary attributes), a user can realize this easily by setting a different edit probability *P* that is non-uniform (e.g., to 70/30 for binary attributes). This may be used for a fairness definition that simply reflects the gender-occupation proportions of current society, i.e., utilizing a country’s occupation statistic a user lives in. This further illustrates that fairness is versatile, and Fair Diffusion is adaptable to different notions. Yet, there is a lot to be done and Fair Diffusion is just one tool in the fairness toolbox.

**Binary gender classification** We acknowledge the limited representation of gender in this study. Current automated measures treat gender as a binary-valued attribute, which it is not [49, 51]. Due to the lack of tools that treat gender beyond the binary, an empirical evaluation at a large scale remains limited to binary-valued gender evaluation. Similarly, Stable Diffusion seems limited to binary gender terms. We observed non-binary gender instructions to result in fragile behavior (cf. Discussion in Appendix C). Although current diffusion models have inherent limitations, Fair Diffusion builds on them to make a first step toward fairness by mitigating, e.g., gender-biased image generation. We advocate for research on treating gender as non-binary in generative and predictive models.

**Technical limitations** Furthermore, we also want to touch upon some technical limitations of this work. First, when evaluating the LAION-5B dataset, we observed that several images are stock photos. This is a reminder that LAION-5B is a web-crawled dataset that does not represent reality, nor does it reflect the internet in its entirety. Second, we use the CLIP encoder for searching LAION-5B. However, as we demonstrated, CLIP is inherently biased [52, 53], which may affect the search results. Therefore, the resulting images are not entirely disentangled from this confounding factor. However, there are barely any alternatives, as manual labeling will be biased too and infeasible due to a large amount of data, and other automated approaches will suffer from imprecision too [54]. We empirically chose the threshold $\delta$ to be low enough to counteract this behavior, so images of disadvantaged genders will be included in the search result. Third, the gender classification results rely on a pre-trained classifier, FairFace [48]. As said, the classifier is an inherent limitation for classifying (binary) gender. Furthermore, we cannot guarantee that this classifier is bias-free and hence promote investigating its function and alternative ways. Yet, it seems to be the best available choice for an automated evaluation. Importantly, FairFace and the other limitations are only relevant to evaluate Fair Diffusion, while the strategy itself is independent of, e.g., the used classifier for evaluating. Lastly, Fair Diffusion currently builds on Sega and inherits its constraints. This is inherent to all currently available approaches that enable editing generative DMs. Fair Diffusion is agnostic to that, and can, in principle, be combined with any editing technique.

**Beyond text-guided fairness** Fair Diffusion currently operates on the textual interface to steer image generation but is not limited to this modality. Fair Diffusion adds fair guidance to the image generation according to edit encodings $c_{e_i}$. Currently, CLIP’s text encoder embedded the edit prompt $e_i$ to $c_{e_i}$. The way the encoding is obtained can go beyond (English) natural language. Fair Diffusion can be extended with other approaches like AltCLIP [55] for multilingual encodings, Textual Inversion [56] for visual encodings, or MultiFusion [57] for multimodal (text and image) encodings. These offer versatile interfaces for fair guidance with Fair Diffusion.

**The challenges of user interaction** While human interaction has generally proven to be helpful [10, 11, 34, 35], at the same time, certain dangers can arise. For example, a user in control with malicious intentions could target the model to misuse it. Like many other pieces of research, Fair Diffusion faces the dual-use dilemma. The strategy can be used in an adversarial manner as well, such that biased outcomes of generative models can be further amplified, and its diversity decreased. Hence, further detection mechanisms for malicious interaction are required. This is an active research topic [58] that needs consideration when using human interaction.

Our work is of greater relevance as it offers the opportunity to immediately promote fairness in many real-world applications. As image generation models become increasingly popular and integrated into our lives, fairness must be kept in mind. DMs come into play even in high-stakes applications such as medicine and drug development [59]. These models are also used in other areas, such as advertisement or design.^11^ Imagine a firefighter advertisement^12^ containing people from Fig. 1 top or bottom row only. This way, generative models can have a crucial impact on societies and how we include and value diversity in them. Furthermore, Fair Diffusion can make another step toward fairness in society. This work focuses on a specific definition of fairness for evaluation purposes. However, the way such a tool is used also has a political dimension beyond research. Sometimes, the goal is not to achieve an equal outcome for each attribute. Temporarily over-representing a certain attribute, e.g., in advertisements, can be desired as it can promote awareness and transparency for bias and discrimination concerning this attribute. Or, current over-representations can be gradually reduced, to slowly habituate new proportions in a society. The pathway toward an ideal discrimination-free world may take measures that might contradict the fairness definition used in this work (Definition 1) but align with other fairness definitions [40]. Hence, our approach facilitates flexible outcome proportions, which, in turn, enables over-representation or any other proportion. Along these lines, we do not argue for a specific proportion or promote a specific political direction. Instead, we provide a strategy that can be used by society and politics immediately with ease for purposes that can ultimately promote a fairer depiction of society. Therefore, the overall goal might not be a fair tool itself, as it is rather a means to an end, but to use it in a way that promotes a fairer society without discrimination.

## Conclusion

In this work, we introduced Fair Diffusion and demonstrated that it can instruct generative text-to-image models in terms of fairness. To this end, we first audited for fairness by exploring the publicly available large-scale training dataset of Stable Diffusion (LAION-5B). We further applied the iEAT to its underlying pre-trained representation encoder (CLIP). Both showed severe gender biases in the downstream diffusion model. Surprisingly, we found gender bias amplification between the data and output distribution for the majority of occupations. However, the diffusion model’s textual interface and advanced steering approaches provide the necessary control to instruct it on fairness, as our extensive evaluation demonstrates. Specifically, we showed how to shift the bias in generated images in any direction yielding arbitrary proportions for, e.g., gender and age. In this way, our method prevents diffusion models from implicitly and unintentionally reflecting or even amplifying biases. Based on our findings, we strongly advise careful usage of such models. However, we also envision easily accessible generative models as a tool to amplify fairness, i.e., itself introducing syntactic biases—compared to real-world distributions—into realistic images. This enables media to display various genders in stereotypically over-represented occupations motivating younger people to follow their interests despite societal biases [60–62].

An exciting avenue for future work is disentangling the components to pinpoint the sources of bias in the model. In addition, this work can be extended to image-to-image diffusion, facilitating the editing of real-world images rather than just generated ones. Lastly, Fair Diffusion can be easily integrated into any real-world diffusion application, mitigating unfair image generation or even amplifying fairness.

## Figures and Tables

**Fig. 8:**
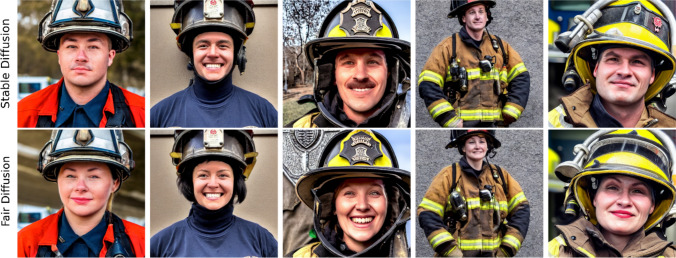
Generated images with SD (top row) and Fair Diffusion (bottom row) for occupation “firefighter”. The images are generated with the prompt “A photo of the face of a firefighter”. We applied fair guidance with − “male person” + “female person”. One can observe that Fair Diffusion changes the typical gender appearance for each occupation image while keeping the residual (occupation-related) features present

**Fig. 9:**
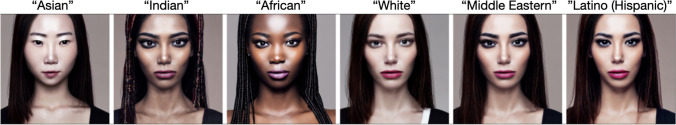
Generated images with Fair Diffusion for “A photo of a woman”. The images are instructed with + “Asian”, + “Indian”, + “African”, + “European”, + “Middle Eastern”, and + “Latino (Hispanic)”. As a consequence, one can observe a shift in outward appearance. This way, generated images are of a broader ethnic diversity with more diverse skin tones

**Fig. 10:**
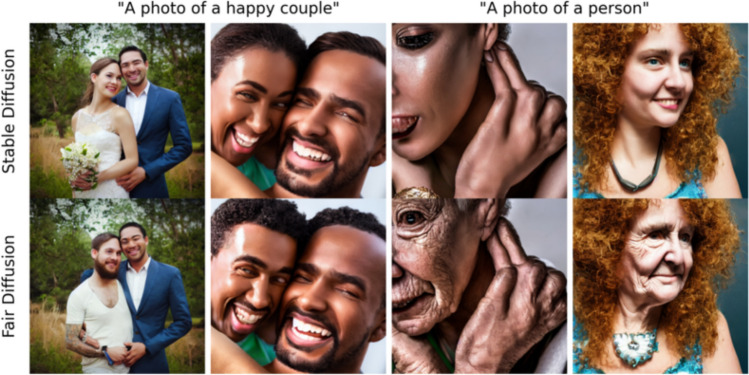
Fair Diffusion approaches heteronormativity or ageism. The top row is generated with default SD and the bottom row with Fair Diffusion. The first two columns are generated with “A photo of a happy couple” and instructed with − “heterosexual” + “homosexual” and the left two images are generated with “A photo of a person” and instructed with − “young person” + “old person”. As one can observe, the outward appearance of the generated persons changes according to the instructions used

**Fig. 11:**
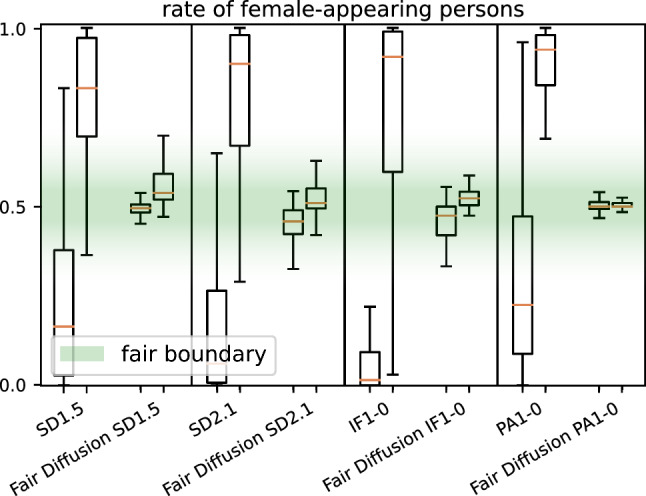
Comparison of Fair Diffusion applied to different models. Per-group (f/m) median rate of female-appearing persons for 150 occupations. Here, Fair Diffusion is applied to 4 different models (SD1.5, SD2.1, IF1-0 and PA1-0). We compare the default model outcome with the outcome when Fair Diffusion is applied. As can be seen, Fair Diffusion successfully mitigates gender-occupation biases on all evaluated models. (Best viewed in color) (color figure online)

**Fig. 12:**
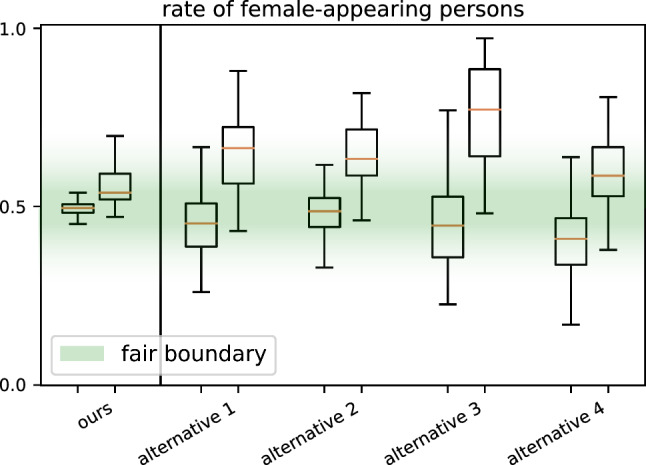
Per-group (f/m) median rate of female-appearing persons for 150 occupations. Here, Fair Diffusion is realized with the 5 different setups shown in Table 3. All alternatives perform worse than our setup, used in Fig. 5. (Best viewed in color) (color figure online)

**Fig. 13:**
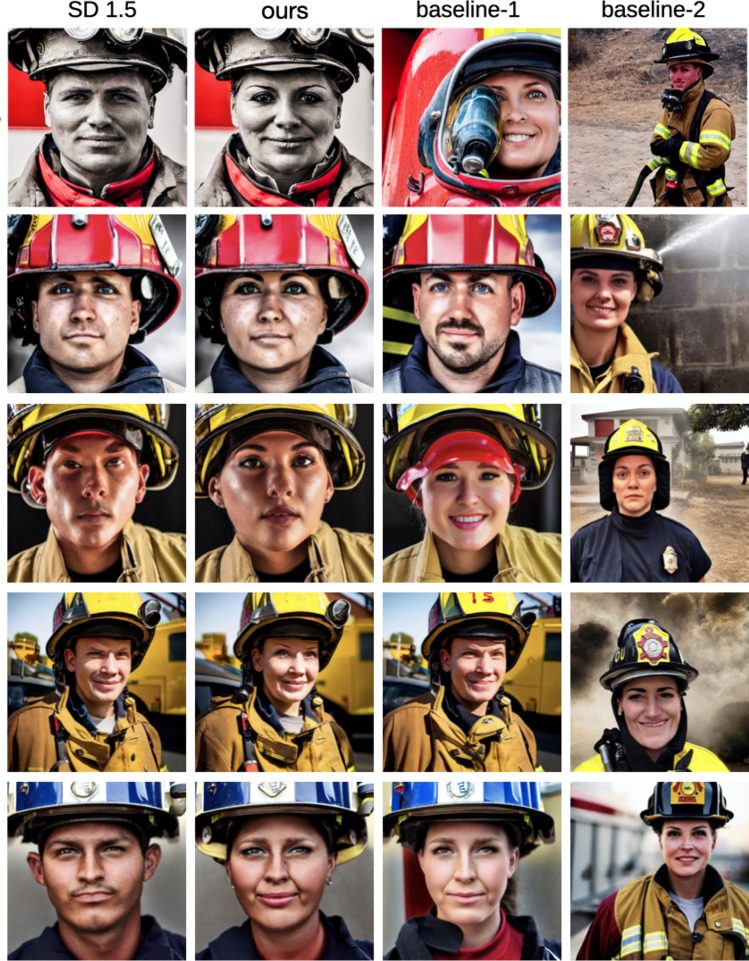
Qualitative comparison of different image editing techniques. The leftmost image is generated by SD 1.5 with the prompt “A photo of the face of a firefighter”. To address the present gender occupation bias, we compare three methods: the two most promising methods from Bansal et al.  [32], i.e., baseline-1 (“A photo of the face of a female firefighter”) and baseline-2 (“A photo of the face of a firefighter if all individuals can be a firefighter irrespective of their gender”), and ours (Sega, increasing “female” and decreasing “male”). As can be seen, the baselines often completely change the image composition and even fail to change gender appearance. Ours, in contrast, prevails the image composition while successful and at the same time limiting the edit to the relevant image region only

**Fig. 14:**
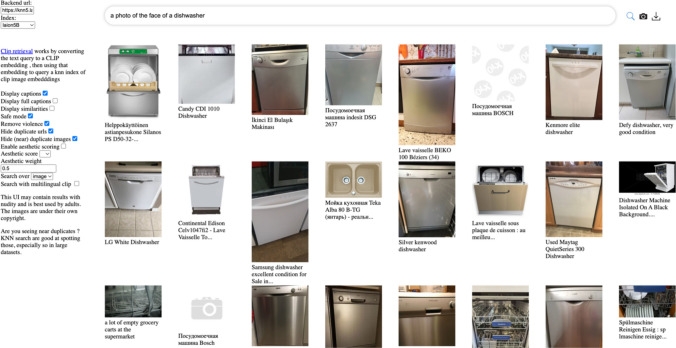
Dishwasher artifact as lack of training data in LAION-5B and the ambiguity of the term “face of a dishwasher”

## Data Availability

All datasets used in this work are publicly available. The prompt set used to generate images is available at https://github.com/ml-research/Fair-Diffusion/blob/main/occupations.txt and https://huggingface.co/spaces/societyethics/DiffusionBiasExplorer/blob/main/promptsadjectives.csv. The LAION-5B dataset is available at https://laion.ai/blog/laion-5b and https://huggingface.co/datasets/danielz01/laion-5b.

## Appendix

### A Further applications and results with Fair Diffusion

Apart from the results shown in the main text, we also generated more images, to provide further insights into Fair Diffusion. In this section, we show further applications and qualitative results about gender, racial, sexual, and age discrimination.

Figure 8 shows again that generated images of “firefighters” by default SD (top row) are strongly male-biased. In contrast, Fair Diffusion changes the outward appearance towards female-appearing “firefighters”. More interestingly, the changes in gender appearance do not change the overall image composition and the occupation remains identifiable. This keeps confounding changes minimal. We regard this as a very powerful property of our approach.

Furthermore, in Fig. 9, we show results for the prompt “A photo of a woman”. Usually, the generated images by default SD represent people with Caucasian appearance. Here, we generated images with Fair Diffusion by instructing with different ethnicity descriptions, based on FairFace [48], which in turn is based on descriptions of the U.S. Census Bureau. In fact, we use + “Asian”, + “Indian”, + “African”, + “White”, + “Middle Eastern”, and + “Latino (Hispanic)”. One can observe that the outward appearance changes according to the instruction given. This way, generated images are of a broader ethnic diversity with more diverse skin tones. Although racial appearance must be used very carefully, Fair Diffusion is a first step in diversifying the generated images of Stable Diffusion. Interestingly, one can observe that the instruction refers to multiple features, like hair color and style, lip color, and shapes of nose, cheek, and chin. This figure illustrates the potential capabilities of Fair Diffusion and should motivate further research in more diverse image generation beyond the presented gender biases.

We found further discriminating behavior in default SD, shown in Fig. 10. As one can observe, SD tends to generate images in line with heteronormativity and images of younger persons. Instead, Fair Diffusion can be employed again to generate homosexual couples and people of different ages. Please note that these images are only illustrative to show the potential of Fair Diffusion. As elaborated in Sect. 3.1, certain dangers also come along. For example, “homosexual” and “gay” often generated male-homosexual couples. This might be due to the fact that there is a specific word for female homosexuality, i.e., lesbian, and with + “lesbian”, it is also possible to generate female couples.

All the results shown are preliminary, pinpointing avenues for future research.^13^

**Table 2:** Bias inspection for CLIP

Topic	Target concept	Attribute concept	$n_t$	$n_a$	p	d
Gender	Male–Female	Career–Family	40	21	0.01	0.58
Gender	Male–Female	Science–Arts	40	21	0.003	0.63
Gender	Male–Female	Engineering–Caregiving	40	12	0.005	0.57
Ethnicity	White Male–Black Female	Career–Family	3	21	0.1	0.99
Ethnicity	White Male–Black Female	Science–Arts	3	21	0.05	1.48
Ethnicity	White Male–Black Female	Engineering–Caregiving	3	12	0.05	1.57
Ethnicity	Other people–Arab-Muslim	Pleasant–Unpleasant	10	55	0.002	1.18
Ethnicity	European American–African American	Pleasant–Unpleasant	6	55	0.002	1.66

We examine the iEAT for gender occupation biases. The table shows that such biases are present in CLIP, i.e., male-appearing persons are considered to be closer to career, science, or engineering compared to female-appearing who are closer to family, arts, and caregiving. All examples have a high effect size, *d*, along with high significance, i.e., low *p* value. Furthermore, we evaluated intersectionality biases and found that skin color attributes amplify gender occupation biases. Lastly, broader ethnicity (cultural) biases can be observed for (un)pleasant. $n_t$ and $n_a$ denote the number of images evaluated for each concept

### B CLIP biases: a more detailed inspection

In this experiment, we tested the similarity between images of different concepts. In line with Steed et al. [47], we applied their setup to the CLIP encoder and extended their occupation experiments with engineering and caregiving. To this end, we added the first 12 images from Google search, that did not contain people, to the set of evaluation images from Steed et al. [47]. All images can be found in our code base^14^ to reproduce the results (Fig. 11).

Besides the results shown in Table 1 in the main text, we here show further results in Table 2. One can observe further cultural and racial biases for images depicting people looking Arab-Muslim or African-American as CLIP relates them to unpleasant. Accordingly, an intersectionality bias [50] is present in CLIP too, amplifying the gender occupation bias. In other words, in CLIP, some people are confronted with multiple factors of advantage (e.g. White male) or disadvantage (e.g. Black female). In the standard setup (first three rows), we compared images of men and women (of all skin tones) to e.g. career and family. In the intersectionality setup (all other rows), we picked a subset, e.g. only White men and Black women, and conducted the same test. One can observe that the bias gets stronger when using these subsets. Overall, we find that CLIP is inherently affected by bias too, and can be attributed as a source for the bias shift between LAION-5B and SD.

### C Ablation of different setups for Fair Diffusion

In addition to the results shown in Fig. 5, we investigated (i) different underlying models and (ii) different prompt setups for Fair Diffusion.

For (i), we compare Fair Diffusion when applied to different models. Next to Stable Diffusion 1.5, we here further examine the top-tier publicly available models Stable Diffusion 2.1 (SD2.1)^15^ [1], DeepFloyd IF 1-0 (IF1-0)^16^ and Paella 1-0 (PA1.0)^17^ [63]. To this end, we implement Fair Diffusion with these underlying models and make the code publicly available. We chose these models as they entail various differences to SD1.5; SD2.1 is a more advanced version of the same model, IF1-0 is a diffusion model on pixel level (in contrast to SD1.5 which does an additional latent space transformation in between), and PA1-0 is an even more different architecture than standard diffusion models operating on quantized space and noised tokens (we refer the interested reader to their manuscript). As we show in Table 11, Fair Diffusion performs well in mitigating gender-occupation biases on all models. The gender-occupation bias in each default model is quite high as the first two boxplots of each block show. With Fair Diffusion we use fair guidance to promote a fairer outcome, i.e. reduce gender-occupation bias. This emphasizes the versatility of Fair Diffusion again as it architecture-agnostic.

For (ii), we show a general setup of the investigated prompt setups in Table 3. The edit instructions used in the main text (denoted as *ours* in the table) were determined by examining a variety of other prompts. We were specifically interested in evaluating more gender-neutral terms. The result can be found in Fig. 12. The applied edit instructions in Fig. 12 correspond to the ones given in Table 3. In general, none of the other four setups achieves average scores for both groups within the fair boundary. Still, these different guiding prompts help mitigate gender-occupation bias compared to standard SD.

**Table 3:** Prompts used for fair guidance

		$e_1$	$e_2$
Ours	f	− “male person”	+ “female person”
	m	− “female person”	+ “male person”
Alternative 1	f	− “male person, female person”	+ “female person”
	m	− “male person, female person”	+ “male person”
Alternative 2	f	–	+ “female person”
	m	–	+ “male person”
Alternative 3	f	− “gender”	+ “female gender”
	m	− “gender”	+ “male gender”
Alternative 4	f	− “female person, male person, non-binary person”	+ “female person”
	m	− “female person, male person, non-binary person”	+ “male person”

In this work, we investigated 5 different setups. As we did binary fair guidance (concepts $e_1$ and $e_2$), each consists of a female (f) and male (m) setup. ‘-’ denotes that a concept is decreased/suppressed during generation and ‘+’ denotes that a concept is increased/promoted during generation

Let us investigate the reasons for this loss in more detail. For one, the measured rate of female-appearing persons depends on the FairFace classifier. In case the edit instructions led to less clearly identifiable generated persons, FairFace struggled to classify them correctly and had higher uncertainty. Alternative 2 uses positive guidance only and shows to be a good way to increase fairness already, while negative guidance helps further improve. On the other hand, alternative 1 shows that positive and negative guidance can interfere with each other, demonstrating the need for distinct guidance concepts to optimize performance. Furthermore, the success of the edit instruction depends on the parameters used. We used rather low parameters to not alter the image too strongly. If one increases the parameters, the changes are enforced stronger, at the expense of more substantial changes to the re-generated image. On the other hand, the instructions given to Fair Diffusion have to be known by the model. SD seems to have only a little understanding of, e.g., the words “gender” (cf. alternative 3) and “non-binary” (cf. alternative 4). Thus, it is difficult to appropriately use these concepts for steering the image toward fairer outcomes. More research is needed here to investigate these findings further.

**Comparing different image editing techniques** In Fig. 13, we compare three different image editing techniques for diffusion models. Fair Diffusion is agnostic to the underlying method and able to integrate various approaches—thus also the three shown. We generate images for “A photo of the face of a firefighter”. Next to Sega (ours), we investigate two prompt mitigation strategies, the two most promising methods from Bansal et al.  [32]. To this end, we use baseline-1 (“A photo of the face of a female firefighter”) and baseline-2 (“A photo of the face of a firefighter if all individuals can be a firefighter irrespective of their gender”). This qualitative comparison shows the impact of different mitigation strategies. Ours is the only one to consistently be successful in realizing the edit while preserving the overall image composition and limiting the edit to the relevant image region only. baseline-1 fails in approaching the gender appearance (rows 2 and 4) and makes unnecessary changes to the background and image features (changes to helmet, clothes, etc.). The result for baseline-2 is even worse. While the method fails in approaching the gender appearance (row 0) too, it further yields completely different images. This way, both baseline methods increasingly run the risk of introducing confounding factors (e.g. gender stereotypes).

**Table 4:** Setup for multi-ary ageism mitigation with Fair Diffusion

		$e_1$	$e_2$	$e_3$
Ours	0–19	− “old person”	− “middle-aged person”	+ “child, young person”
	20–39	− “child, young person”	− “old person”	+ “middle-aged person”
	40–	− “child, young person”	− “middle-aged person”	+ “old person”

We show the concepts which are decreased (–; $e_1$ and $e_2$) and increased (+; $e_3$) for each of the three age groups

### D Further experimental details

Before we explain further experimental details, we describe the number of evaluated and generated images.

Overall, we evaluated 2.32M images in this work, of which 0.49M are generated images and 1.83M are LAION images. In more detail, for Fig. 5, we identified 1.83M LAION gender-occupation images and generated 37,500 with SD and 37,500 with Fair Diffusion. In Fig. 7, we applied Fair Diffusion to ageism and generated another 37500. Moreover, we ablated Fair Diffusion with 4 different prompts (Fig. 12), and generated another $4\times 37500$ images. Furthermore, we evaluated 3 other models for gender-occupation bias and their mitigation (Fig. 11), i.e., $3\times 2\times 37500$ images. This way, we generated $37500+37500+37500+4\times 37500+3\times 2\times 37500=13\times 37500=487500$ images and thus evaluated $1.83\text {M}+0.49\text {M}=2.32$M images.

**Implementation details** For assessing the bias of text-to-image DMs and its mitigation, we inspect the publicly-available diffusion model Stable Diffusion v1.5 (SD [1]),^18^ its underlying large-scale dataset (LAION-5B [12])^19^ and pre-trained model (CLIP [13]).^20^ Our instruction tool is built around Sega^21^ to edit images and guide the image generation toward fairer outcomes and employed FairFace^22^ [48] as $\kappa$ to derive the protected attribute, i.e. facial (gender) attributes.

**Multi-ary** Fair Diffusion Similar to fair guidance for binary gender, we conditioned the image generation with Fair Diffusion now applied to ageism. Figure 7 showed the results for this experiment in which Fair Diffusion clearly improved on default biases of SD1.5 toward the fair boundary. Here, we explain the setup in more detail. We discretized age into three groups (0–19, 20–39, 40-), which we show with their respective guidance concepts in Table 4. In this setup, we simultaneously steered away from two concepts (two age groups; $$e_1$$ and $$e_2$$) and guided toward the desired age group ($$e_3)$$. According to Definition 1, we chose one of the three rows with a probability of $$P=1/3$$ during image generation. This way, we utilize the concepts encoded in a DM to simultaneously suppress one and reinforce the others, with alternating directions. Due to this approach, 1/3 of the images should represent “0–19/20–39/40–”-appearing generated persons.^23^ We applied Fair Diffusion to SD-generated images, i.e., we re-generated an image with the same seed and parameters and included the additional fair guidance $$\gamma$$ for age. We evaluated the generated images with FairFace’s age classifier.

**Artifacts in image generation** As described in our experimental evaluation, we also stumbled upon some challenges. For example, it was difficult to generate images for the occupation “dishwasher”. In Fig. 14, one can observe that “face of a dishwasher” is very ambiguous and mainly yielded results of the front side of dishwashing machines. Hence, further prompts beyond “A photo of the face of a {occ}” should be evaluated in future research.

**Selection of subgroups** We hand selected subgroups “science”, “arts”, “engineering”, and “caregiving”. The selection can be found in Table 5.

**Table 5:** Hand-selected subgroups of the whole occupation list

Science	Arts	Engineering	Caregiving
Aerospace engineer	Artist	Aerospace engineer	Childcare worker
Claims appraiser	Author	Architect	Coach
Clerk	Designer	Civil engineer	Dental assistant
Computer programmer	Interior designer	Computer programmer	Dental hygienist
Electrical engineer	Musician	Computer support specialist	Dentist
Scientist	Painter	Computer systems analyst	Doctor
	Photographer	Electrical Engineer	Housekeeper
	Singer	Engineer	Maid
	Writer	Industrial engineer	Massage therapist
	Graphic designer	Mechanical engineer	Mental health counselor
		Programmer	Nurse
		Software developer	Nursing assistant
			Occupational therapist
			Physical therapist
			Psychologist
			Social assistant
			Social worker
			Teacher
			Teaching assistant
			Therapist

The list is divided into four subgroups: Science, Arts, Engineering, and Caregiving. Each subset contains between 50K and 120K images

## Footnotes

1 Available at https://anonymous.4open.science/r/faidi_anon-7D6E.

2 There are also other post-process methods available, e.g. prompt engineering methods [32], but they substantially change the generated content.

3 Taken from https://huggingface.co/spaces/society-ethics/DiffusionBiasExplorer.

4 We explain the exact number in more detail in Appendix D.

5 Selection is explained in Appendix D.

6 The share of images classified by $\kappa$ (Eq. 7) as “female”-appearing.

7 We denote a reflection if the SD outcome bias corresponds to the LAION-5B bias $\pm 4\%$, and an amplification (mitigation) if the bias is farther away from (closer to) the fair boundary.

8 In total, 64 of 150 occupations are more female-biased, while 86 are more male-biased.

9 We applied the chosen guidance to the image generation regardless of its outcome without guidance or any biases present in LAION-5B.

10 https://knn5.laion.ai/.

11 https://www.cosmopolitan.com/lifestyle/a40314356/dall-e-2-artificial-intelligence-cover/.

12 For example, stock images are already being generated using diffusion models (Shutterstock, https://www.shutterstock.com/press/20435).
